# Mitochondrial structure alteration in human prostate cancer cells upon initial interaction with a chemopreventive agent phenethyl isothiocyanate

**DOI:** 10.1186/1475-2867-14-30

**Published:** 2014-03-31

**Authors:** Chengsen Xue, Hilda A Pasolli, Irene Piscopo, Daniel J Gros, Christina Liu, Yamei Chen, Jen Wei Chiao

**Affiliations:** 1 Department of Medicine, New York Medical College, Valhalla, NY 10595, USA; 2 ICON Central Laboratories, Farmingdale, NY 11735, USA; 3 The Rockefeller University, 1230 York Avenue, New York, NY 10021, USA; 4 EM Consulting, Huntington, NY 11743, USA; 5 Thomson Reuters, 1 New York Plaza, New York, NY 10004, USA

**Keywords:** Phenethyl isothiocyanate, PEITC, Mitochondria, Prostate, Organelle target

## Abstract

**Background:**

Phenethyl isothiocyanate (PEITC), present naturally in cruciferous vegetables, is a chemopreventive agent. It blocks initiation and post-initiation progression of carcinogenesis. Mechanism study in human prostate cancer cells revealed that PEITC is a dual inhibitor of aberrant DNA hypermethylation and histone deacetylases, reactivating silenced genes and regulating the androgen-mediated growth of tumor cells. The identity of the cellular organelle that initially interacts with PEITC has not been fully described.

**Methods:**

Human prostate cancer LNCaP cells were exposed to PEITC and the effects on cellular fine structure examined by transmission electron microscopic studies. Alteration of mitochondrial membrane potential and cytochrome c release were evaluated as early events of apoptosis, and the TUNEL method for quantifying apoptotic cells. Mitochondria were isolated for determining their protein expression.

**Results:**

Ultrastructural analyses have revealed condensed mitochondria and a perturbed mitochondrial cristae structure, which assumed a rounded and dilated shape within 4-hours of PEITC contact, and became more pronounced with longer PEITC exposure. They presented as the most prominent intracellular alterations in the early hours. Mitochondria structure alterations were demonstrated, for the first time, with the isothiocyanates. An increase in the number of smooth endoplasmic reticulum and vacuoles were also noted that is consistent with the presence of autophagy. Early events of apoptosis were detected, with cytochrome c released along with the appearance of mitochondrial alteration. Mitochondrial membrane potential was disrupted within 18 hours of PEITC exposure, preceding the appearance of apoptotic cells with DNA strand breaks. In parallel, the expression of the mitochondrial class III ß-tubulin in the outer membrane, which associates with the permeability transition pore, was significantly reduced as examined with isolated mitochondria.

**Conclusion:**

Mitochondria may represent the organelle target of the isothiocyanates, indicating that the isothiocyanates may be mitochondria-interacting agents to inhibit carcinogenesis.

## Background

Among the well-documented chemopreventive agents are the isothiocyanates, which occur naturally in a wide variety of cruciferous vegetables including broccoli, cabbages, kale, cauliflower, watercress, and mustard [1]. When the vegetables are cut or masticated, the enzyme myrosinase is released to hydrolyze glucosinolates, which yield isothiocyanates [2]. Phenethyl isothiocyanate (PEITC) is derived from the hydrolysis of the glucosinolate gluconasturtin, which is found in high amounts in watercress [3,4]. In the studies of prostate cancer, PEITC has been demonstrated as a dual inhibitor of DNA hypermethylation and histone deacetylases, reactivating silenced genes including the π-class glutathione S-transferase which is inactivated by aberrant DNA methylation in the vast majority of clinical prostate tumors [5]. PEITC and its major metabolite have been demonstrated to inhibit post-initiation progression of carcinogenesis, including the induction of autophagy, apoptosis and down regulation of the androgen receptor thus reducing its growth stimulation on prostate tumor cells [6-8]. Some isothiocyanates, including PEITC, bind covalently to cysteines in tubulin as a molecular target, which may mediate microtubule disruption and blocking mitosis [9]. The identity of the cellular organelle as the target in the initial interaction with PEITC, have not been fully described.

Mitochondrial and ribosomal dysfunctions have been implicated in cancer [10-13]. The implication includes an impaired respiratory capacity with increased utilization of glucose, which is considered as a hallmark of cancer [14,15]. This may in part be related to the structure of mitochondria, such as the surface area of the inner membrane and number of cristae, which are correlated with the aberrant activities of metabolic enzymes including the oxidative phosphorylation and cytochrome c oxidase [16-19]. Aberration of mitochondria-mediated apoptosis in cancer cells has been described. An example is the mitochondrial membrane potential that is related to apoptosis initiation is known to be higher in cancer than in normal cells [20,21]. Novel agents targeting mitochondria and their pathways in malignant cells are being explored [22-26].

The structure of mitochondria consists of an outer membrane wall, and the internal matrix space is partitioned with multiple cristae which are compartments wrapped by an inner membrane. There are multiple protein complexes in the membrane and cristae mediate the mechanisms of cellular energy and cell death. This presentation describes the identification of the alterations in mitochondrial structure and functions mediated by PEITC through ultrastructural and biochemical analyses. Mitochondria may represent an early organelle target in the interaction with isothiocyanates to inhibit carcinogenesis.

## Results

### Mitochondrial structure alteration by an isothiocyanate

**Figure 1:**
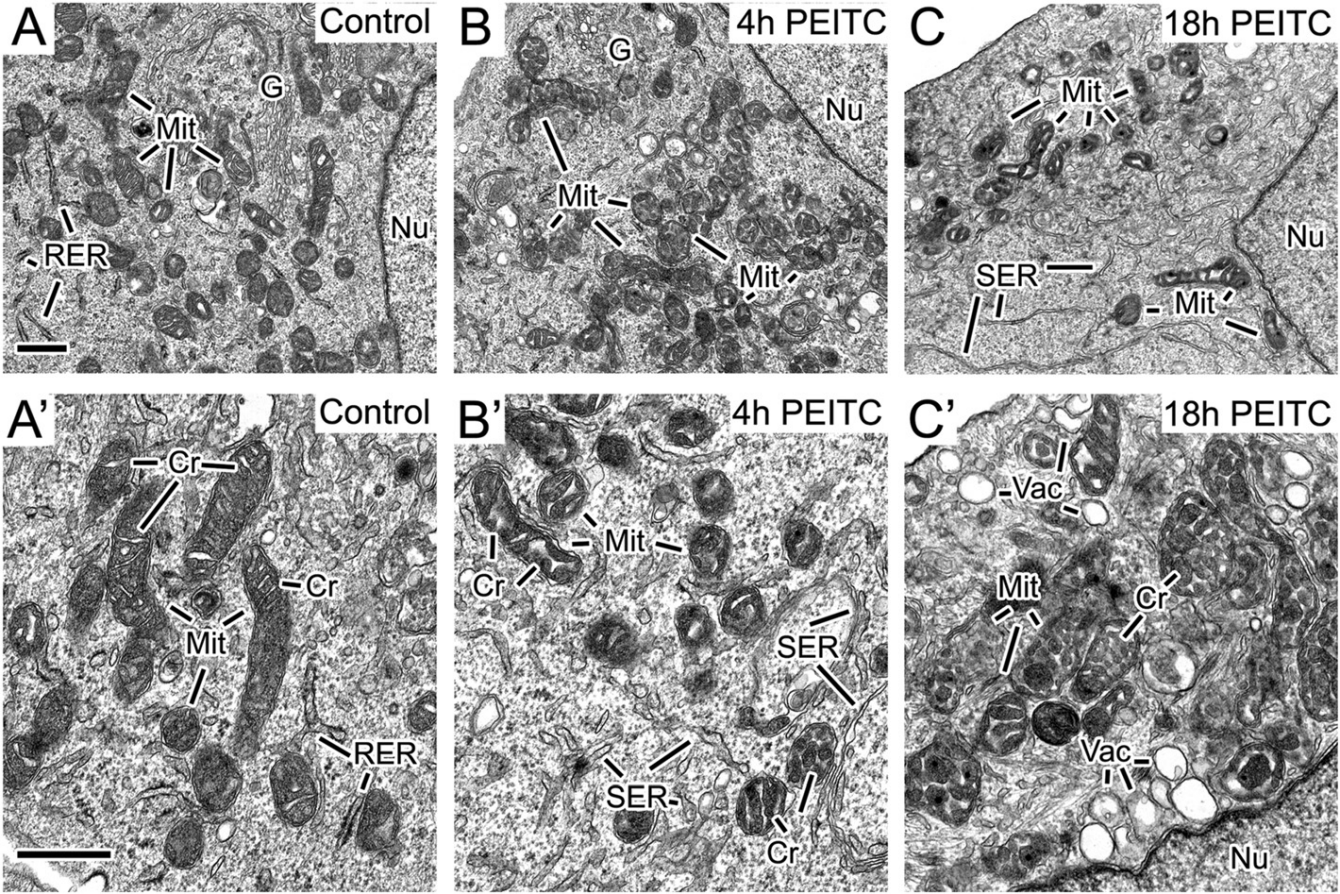
**Alteration of mitochondria structure by PEITC.** Alteration of cellular fine structure in human prostate cancer cells LNCaP exposed to PEITC. LNCaP cells without or with the exposure to PEITC at 8 μM were examined by transmission electron microscopy at 4 and 18 hours. Frame **A** and **A**’ show the control cells without the PEITC. Frames **B-B**’ and **C-C**’ depicted respectively the PEITC-exposed cells for 4 and 18 hours. Mit: mitochondria; G: Golgi; Nu: nucleus; RER: rough endoplamic reticulum; SER: smooth endoplasmic reticulum; Cr: cristae; and Vac: vacuoles. Bar in **A** = 0.5 μm, valid for **B** and **C**. Bar in **A**’ = 0.5 μm, valid for **B**’ and **C**’.

A human prostate cancer cell line LNCaP was chosen for examining the effects of PEITC on cellular organelles since its cellular growth dependents on androgen, and the inhibitory effects of the PEITC has been demonstrated. To evaluate the early effects of PEITC on the fine structure of prostate cancer cells, untreated LNCaP cells and those exposed to 8 μM PEITC for 4–18 hours were prepared for transmission electron microscopic examination. In comparison to the untreated cells (Figure 1A and A’), the main change in the PEITC-exposed cells was that they exhibited a perturbed mitochondrial cristae structure and condensed mitochondria. The cristae adopted a rounded and dilated shape, which was observed after 4 hours of PEITC exposure (Figure 1B and B’), and became progressively more pronounced at 18 hours of treatment (Figure 1C and C’). The condensed mitochondria appeared as dense bodies with a dark matrix and translucent cristae, which were present after 4 hour (Figure 1B and B’) and 18 hour PEITC treatment (Figure 1C and C’). At 4 hours the PEITC-exposed cells also showed more smooth endoplasmic reticulum and vacuoles in the cells (Figure 1B’ and C’) as compared to the control. The vacuoles with larger sizes were more pronounced after 18 hours. There was no observable difference in the nuclei ultrastructure between control and the treated cells. Both of them displayed abundant euchromatin without much heterochromatin. The results have indicated that the mitochondria were the organelle with the most prominent alterations at early hours upon interacting with PEITC. Alteration of mitochondria structure including cristae remodeling occur during the early stages of apoptosis [27,28]. Events of apoptosis were subsequently examined during the early hour of interaction with PEITC.

### Release of cytochrome c

**Figure 2:**
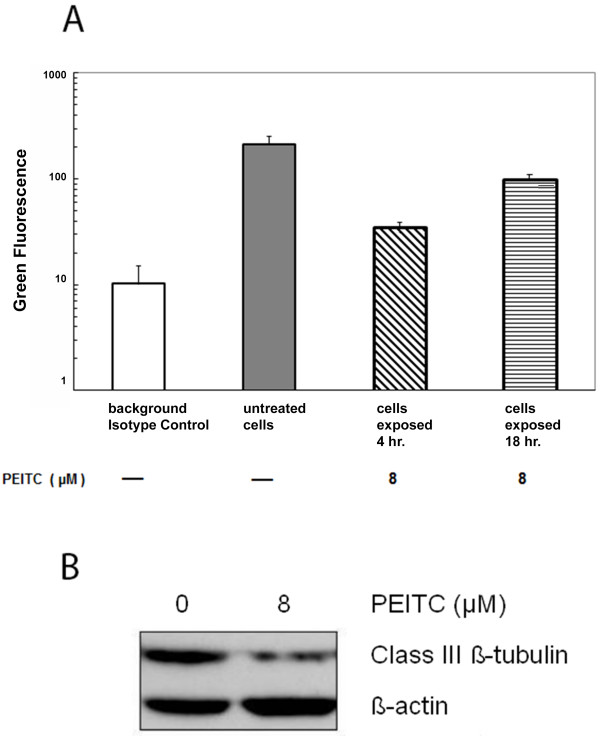
**Decrease of mitochondrial cytochrome c level and class III ß-tubulin by PEITC.** Graph 2**A** shows mitochondrial cytochrome c levels after the LNCaP cells exposed to 8 μM of PEITC for 4 or 18 hours. □ indicates the background FITC-fluorescence level, in arbitrary log unit, which were from cells stained with an isotype control instead of the cytochrome c antibody. ▒ indicates the cytochrome c level in mitochondria of untreated LNCaP cells, which was a mean of the LNCaP cells cultured for 4 and 18 hours. ▧ indicates a reduced FITC-cytochrome c level in mitochondria after the cells were exposed to 8 μM PEITC for 4 hr, and ▤ indicates the cytochrome c level at 18 hr. Vertical bars indicate the ranges of the three highest peaks of fluorescence obtained in each experimental condition. Western blot in Figure 2**B** shows the down-regulation of the mitochondrial class III ß-tubulin protein expression in the isolated mitochondria preparation from the LNCaP cells, which were treated with 8 μM PEITC for 18 hours. The mitochondria preparation isolated from untreated LNCaP was used as a control for comparison. The levels of ß-actin were assayed as a loading control.

We next examined the relation of the altered mitochondrial structure with cytochrome c release, which is known as an early event of apoptosis. The level of cytochrome c in mitochondria of LNCaP cells were determined after exposure for 4 or 18 hours to 8 μM PEITC, or to a vehicle control employing a Millipore cytochrome c assay kit. The mitochondria was permeabilized allowing a FITC-fluorescence labeled anti-cytochrome c antibody to react with cytochrome c in mitochondria, and the fluorescence measured by flow cytometry. Viable cells demonstrate a higher level of cytochrome c staining in mitochondria, while apoptotic cells which have their cytochrome c released from mitochondria to the cytoplasm, have reduced fluorescent staining in mitochondria. The fluorescence level of cytochrome c in mitochondria of untreated LNCaP cells were compared to LNCaP cells exposed to PEITC. Figure 2A shows that both 4- and 18-hour PEITC treatments had reduced mitochondria cytochrome c level than the untreated cells, with the 4 hour-treatment level lower than the 18 hour-treatment. The cytochrome c level in mitochondria, after 4-hour PEITC treatment, was approximately 16.6% of the untreated cell level, while the 18 hours was approximately 47.6% of the untreated cells. As the cytochrome c in mitochondria at 4 hour was less than that of the 18 hour, they indicate that more cytochrome c in mitochondria was released at 4 hour than 18 hour.

### Class III ß–tubulin expression

The mitochondrial class III β-tubulin associates with the voltage-dependent anion channel which is part of the multi-protein permeability transition pore [29]. The expression of the class III ß-tubulin was examined during cytochrome c release, using isolated mitochondria, from cells with or without exposed to 8 μM PEITC for 18 hours. Western blot in Figure 2B depicts that the levels of the class III ß-tubulin in PEITC-exposed mitochondria was reduced approximately 74%, as compared to that without PEITC. The results have indicated that the PEITC treatment down-regulated the expression of the tubulin in the multi-protein permeability transition pore, suggesting a possible association with the alteration of the mitochondria structure and functions.

### Mitochondrial membrane potential disruption

**Figure 3:**
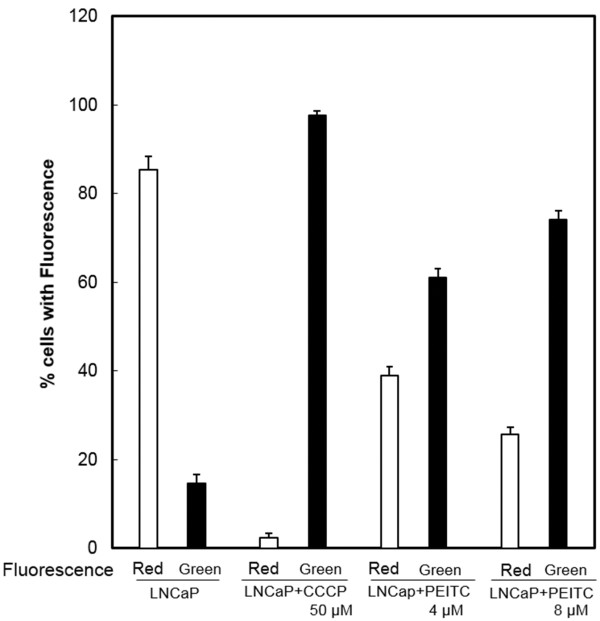
**Disruption of mitochondrial membrane potential.** The LNCaP cells were incubated without or with 4 μM or 8 μM of PEITC for 18 hrs. The cells were then stained with JC-1 dye to measure the membrane potential with a flow cytometric method. Separate control cell cultures were stained with CCCP, a reagent known to disrupt the mitochondrial membrane potential that shifts the untreated cells with red fluorescence to the potential disrupted cells with green florescence. The figure shows that after PEITC exposure there was a concentration-related shift of the LNCaP cells with the red fluorescence to green color as that seen with the control CCCP for membrane potential disruption. (□) indicates the percent LNCaP cells with red fluorescence, and (▐ ) indicates the percent of LNCaP cells with the green fluorescence. The vertical bars indicate the means and ranges of two independent experiments.

A disruption of mitochondrial membrane potential has described as an early event of the apoptosis process [30]. Mitochondrial membrane potential was examined by staining with a dye, JC-1, which forms a color aggregate depending on the membrane potential, and the fluorescence measured by a flow cytometric method. As a positive control, the LNCaP cells were exposed to an agent CCCP that is known to disrupt membrane potential, which resulted in a shift from red fluorescence of untreated cells to green florescence. LNCaP cells were treated with 4 or 8 μM PEITC for 4–18 hours, and a concentration-related shift from red to green fluorescence was observed. Figure 3 shows representative analyses after 18 hours of treatment. Cells exposed to 4 μM PEITC have an increase of green fluorescence from 14.6% of untreated cells, to 61.1% of treated cells, representing approximately four fold increase. When cells were exposed to 8 μM PEITC, the cells with green fluorescence increased from 14.6% of untreated cells to 74.2%, indicating a fivefold enhancement. The results have indicated that the mitochondrial membrane potential was disrupted by PEITC.

Previous reports from us and others have described that PEITC induces growth arrest and apoptosis in human prostate cancer cells, including the LNCaP cell line [5-7,31]. The apoptosis events of the LNCaP cells were therefore evaluated during the early hours of interaction with PEITC, along with the appearance of the alteration of mitochondrial structure. Table 1 describes that apoptotic cells with DNA strand breaks, as enumerated by the TUNEL assay, were mainly present after 18–48 hours of PEITC exposure. The levels of apoptotic cells prior to 18 hours with 8 μM PEITC were close to the background level, the same as the electron microscopic examination which did not observe many apoptotic cells, during the same time period. Since the mitochondrial structure alteration, membrane potential disruption, and cytochrome c release were observed within 18 hours of PEITC exposure, the results indicated that apoptotic cells became detectable after these early events of apoptosis.

**Table 1:** **Percent apoptotic cells of PEITC-exposed prostate cancer LNCaP cells**^**1**^

**PEITC (μM)**	**Exposure time (h)**		
	4	18	48
0	1.3 ± 1.5	1.6 ± 2.1	3 ± 2
5	1.3 ± 1.5	6.5 ± 3.8	24 ± 7.2
8	2 ± 1.7	7.7 ± 2.7	32.3 ± 11.2

^1^Apoptotic cells were determined by the TUNEL method. Numbers ± SE were obtained from separate five experiments.

## Discussion

Ultrastructural analyses have revealed that PEITC affects mitochondria. The cristae developed a rounded and dilated shape within four hours, along with condensed mitochondria representing the most prominent intra-cellular change in the early hours. It is the first time to demonstrate an altered mitochondrial structure by an isothiocyanate. PEITC and other isothiocyanate have been described to mediate growth arrest and apoptosis in different types of cancer cells, and mitochondria may represent the early organelle target of the isothiocyanates to inhibit carcinogenesis.

Parallel to the alteration of mitochondria structure, early events of apoptosis including mitochondrial membrane potential disruption and cytochrome c release were demonstrated. In the subsequent hours, the presence of apoptotic cells with DNA strand breaks could then be detected, which confirms the previously reports of the induction of apoptosis by PEITC [7,31]. Altered mitochondrial structure, such as condensed mitochondria has functional correlate. As it was first characterized in liver mitochondria by electron microscopic examination [32], condensed mitochondria indicate an effort to increase ATP production [33,34]. Our finding of the PEITC-mediated mitochondrial structural alteration and its relation with apoptosis is in line with earlier report that altered morphology and structure of mitochondria were present during early stages of apoptsis [28].

An alteration in cristae shape and surface area signifies changes of protein functions in the cristae compartment, as well as those occurring in the mitochondrial membranes. The changes seen in membrane potential and the class III ß-tubulin may represent, in part, the alterations by PEITC. This is because the inner and outer membranes have other functions. For instance, the inner membrane contains the electron transport-proteins that move metabolites across the membrane, while the outer membrane contains the permeability transition pore, and the voltage-dependent anion channel for molecules traversing through mitochondria. Isothiocyanates might affect functions in metabolism and energy that could be pivotal for tumor cell survival, which are currently being investigated.

Down-regulation of the mitochondria class III ß-tubulin, which locates in the outer membrane, has indicated that PEITC may affect membrane proteins and their functions, and may be involved in the alteration of mitochondria structure. De Gendt et al. [35] reported that the class III ß-tubulin gene is regulated by androgen in mouse and rat sertoli cells, describing it as a direct target of the androgen receptor. Since PEITC is known to reduce the androgen receptor in the LNCaP cells [8], the down-regulation of the class III ß-tubulin could be the result of reduced androgen receptor. Another possibility for the down-regulation of the class III β-tubulin is that tubulin is the molecular target of PEITC [9], and a reduced β-tubulin could be the result of the binding of PEITC to the β-tubulin which is currently being investigated.

Transmission electron microscopy has revealed an increase of the smooth endoplasmic reticulum by PEITC. They are usually prominent in cells specializing in lipid metabolism. For example, hepatocytes have abundant smooth endoplasmic reticulum as they produce lipoproteins particles, and they contain phase 1 enzymes such as Cytochrome P450s to process lipid soluble chemicals and other harmful compounds produced by metabolism. Whether the increase of smooth endoplasmic reticulum associated with the detoxifying activity of PEITC [2,4], and whether PEITC is being metabolized there remains to be investigated. Electron microscopy also detected an increase of vacuoles upon PEITC treatment. They could be due to autophagy which precedes apoptosis. This interpretation is in line with the report of Bommareddy et al. [31] that autophagy could be induced by PEITC in human prostate cancer PC-3 cells.

## Conclusion

Mitochondria have been determined as the primary intracellular organelle of the phenethyl isothiocyanate (PEITC) during the early interaction between the isothiocyanate and the prostate cancer cells. The interaction caused significant structure alteration of mitochondria including perturbed cristae structure with rounded and dilated shape, condensed mitochondria, and a changed expression of mitochondrial class III ß-tubulin which associates with the permeability transition pore. The alteration of mitochondria structure may be involved in the early events of the mitochondrial pathway of apoptosis, indicating the isothiocyanates may be mitochondria-interacting agents.

## Methods

### Cell cultures and reagents

Human prostate cancer cell line LNCaP was purchased from ATCC and characterized there for fewer than 6 months before experimentation. Cells were seeded at 1.5 × 10^5^ cells/ml with RPMI-1640 medium, 10% FCS, and antibiotics. Some cultures were exposed for various time period to 8 μM phenethyl isothiocyanate (PEITC), which was prepared in phosphate buffer and DMSO [5]. A DMSO vehicle control was used in every experiment. PEITC was purchased from LKT Labs with 98% purity. Apoptotic cells were determined by the presence of DNA strand breaks measured by terminal deoxynucleotidyl transferase-mediated biotinylated UTP nick end-labeling (TUNEL) [7].

### Transmission electron microscopy

LNCaP cells were fixed with 2% glutaraldehyde in sodium cacodylate buffer and embedded in Epon resin. Ultrathin sections were cut, collected on 200 mesh copper grids and stained with uranyl acetate and lead citrate. They were examined in the Tecnai G2 Biotwin at 80 kV (FEI). Images were collected with an AMT 2 K digital camera system.

### Mitochondrial membrane potential

Mitochondrial membrane potential was measured with a potential sensitive dye 5,5′,6,6′-tetrachloro-1,1′,3,3′-tetraethylbenzimidazolylcarbocyanine iodide (JC-1) with method described elsewhere [36]. In healthy cells, JC-1 stains the mitochondria red due to the formation of dye aggregates. When the membrane potential is disrupted, the JC-1 dye accumulates in cytoplasm as monomeric form, which shows green fluorescence. The LNCaP cells with or without PEITC exposures were incubated with JC-1 at 10 μg/ml. Cells were washed and the florescence measured with a Becton-Dickinson FACScan flow cytometer. A reagent carbonyl cyanide 4-(trifuoromethoxy)phenyhydrazone (CCCP) that mediates the collapse of membrane potential was added to cells at 50 μM, as a positive control [36].

### Cytochrome c release

The amount of cytochrome c released from mitochondria was evaluated in reference to the level of cytochrome c remained in mitochondria in the experiments. Mitochondrial cytochrome c was determined employing a Millipore’s FlowCellect cytochrome c kit according to manufacturer’s direction. Essentially, cells were incubated for 10 min with a permeabilization solution to achieve selective permeabilization of mitochondria. Cells were fixed, incubated with a blocking buffer, and stained with a FITC-labeled antibody for cytochrome c present in mitochondria. An isotype control immunoglobulin was used in place of the anti-cytochrome c antibody as a background. The fluorescence intensity was measured by a BD FACSCalibur flow cytometer.

### Mitochondria isolation

Mitochondria from LNCaP cells were isolated with a mitochondrial isolation kit (Thermo Scientific) following the manufacturer’s instructions. Essentially, each pellet of 2 × 10^7^ cells was lysed and centrifuged at 12,000 × g to separate the cytosol fraction from the mitochondria. Protein lysates were prepared from the isolated mitochondrial pellets. Western blotting was performed with 10% SDS-PAGE [8]. A polyclonal antibody against the mitochondrial class III ß-tubulin (Covance Research Products) was used for immunoblotting [37]. To ascertain the antibody reacted with the class III ß-tubulin from mitochondria, a purified porin preparation from the mitochondrial membrane which contains class III ß-tubulin (Covance) was used in immunoblotting as a positive control. An antibody against ß-actin was used as a loading control. The percent change of the expression of class III β-tubulin in cells, without or with PEITC treatment, was calculated based on the densities of the tubulin immune complexes from each cell preparations.

## Abbreviations

PEITC: Phenethyl isothiocyanate; CCCP: Carbonyl cyanide 4-(trifuoromethoxy)phenyhydrazone; JC-1: 5,5′,6,6′-tetrachloro-1,1′,3,3′-tetraethylbenzimidazolylcarbocyanine iodide.

## Competing interests

The authors declare that there are no conflicts of interests.

## Authors’ contributions

JWC conceived the study and with CX designed and carried out the investigation. HAP and IP performed the critical electron microscopic studies. DJG, CL, and YC participated in the studies and coordinated the manuscript. All authors read and approved the manuscript.

## References

[B1] Kinghorn AD Su BN Jang DS Chang LC Lee D Gu JQ Carcache-Blanco EJ Pawlus AD Lee SK Park EJ Cuendet M Gills JJ Bhat K Park HS Mata-Greenwood E Song LL Jang M Pezzuto JM Natural inhibitors of carcinogenesis Planta Med 2004 70 8 691 705 10.1055/s-2004-827198 15326546

[B2] Hecht SS Chemoprevention by isothiocyanates J Cell Biochem Suppl 1995 22 195 209 8538199 10.1002/jcb.240590825

[B3] Wattenberg LW Chemoprevention of cancer Cancer Res 1985 45 1 1 8 3880665

[B4] Zhang Y Talalay P Anticarcinogenic activities of organic isothiocyanates: chemistry and mechanisms Cancer Res 1994 54 7 1976s 1981s 8137323

[B5] Wang LG Beklemisheva A Liu XM Ferrari AC Feng J Chiao JW Dual action on promoter demethylation and chromatin by an isothiocyanate restored GSTP1 silenced in prostate cancer Mol Carcinog 2007 46 1 24 31 10.1002/mc.20258 16921492

[B6] Chiao JW Chung F Krzeminski J Amin S Arshad R Ahmed T Conaway CC Modulation of growth of human prostate cancer cells by the N-acetylcysteine conjugate of phenethyl isothiocyanate Int J Oncol 2000 16 6 1215 1219 10811998 10.3892/ijo.16.6.1215

[B7] Chiao JW Wu H Ramaswamy G Conaway CC Chung FL Wang L Liu D Ingestion of an isothiocyanate metabolite from cruciferous vegetables inhibits growth of human prostate cancer cell xenografts by apoptosis and cell cycle arrest Carcinogenesis 2004 25 8 1403 1408 10.1093/carcin/bgh136 15016658

[B8] Wang LG Liu XM Chiao JW Repression of androgen receptor in prostate cancer cells by phenethyl isothiocyanate Carcinogenesis 2006 27 10 2124 2132 10.1093/carcin/bgl075 16704988

[B9] Mi L Xiao Z Hood BL Dakshanamurthy S Wang X Govind S Conrads TP Veenstra TD Chung FL Covalent binding to tubulin by isothiocyanates. A mechanism of cell growth arrest and apoptosis J Biol Chem 2008 283 32 22136 22146 10.1074/jbc.M802330200 18524779 PMC2494917

[B10] Burnett BB Gardner A Boles RG Mitochondrial inheritance in depression, dysmotility and migraine? J Affect Disord 2005 88 1 109 116 10.1016/j.jad.2005.05.009 16019080

[B11] Jiang Y Wang X Comparative mitochondrial proteomics: perspective in human diseases J Hematol Oncol 2012 5 11 23 10.1186/1756-8722-5-11 22424240 PMC3337254

[B12] Li X Zhang K Li Z Unfolded protein response in cancer: the physician’s perspective J Hematol Oncol 2011 4 8 17 10.1186/1756-8722-4-8 21345215 PMC3060154

[B13] Shenoy N Kessel R Bhagat TD Bhattacharyya S Yu Y McMahon C Verma A Alterations in the ribosomal machinery in cancer and hematologic disorders J Hematol Oncol 2012 5 32 40 10.1186/1756-8722-5-32 22709827 PMC3438023

[B14] Hanahan D Weinberg RA Hallmarks of cancer: the next generation Cell 2011 144 5 646 674 10.1016/j.cell.2011.02.013 21376230

[B15] Warburg OH Dickens F The Metabolism of Tumours: Investigations from the Kaiser Wilhelm Institute for Biology 1930 Constable & Company Limited: Berlin-Dahlem

[B16] Capuano F Guerrieri F Papa S Oxidative phosphorylation enzymes in normal and neoplastic cell growth J Bioenerg Biomembr 1997 29 4 379 384 10.1023/A:1022402915431 9387098

[B17] Capuano F Varone D D’Eri N Russo E Tommasi S Montemurro S Prete F Papa S Oxidative phosphorylation and F(O)F(1) ATP synthase activity of human hepatocellular carcinoma Biochem Mol Biol Int 1996 38 5 1013 1022 9132148

[B18] Cuezva JM Ostronoff LK Ricart J Lopez de Heredia M Di Liegro CM Izquierdo JM Mitochondrial biogenesis in the liver during development and oncogenesis J Bioenerg Biomembr 1997 29 4 365 377 10.1023/A:1022450831360 9387097

[B19] Sun AS Sepkowitz K Geller SA A study of some mitochondrial and peroxisomal enzymes in human colonic adenocarcinoma Lab Invest 1981 44 1 13 17 6256583

[B20] Johnson LV Walsh ML Bockus BJ Chen LB Monitoring of relative mitochondrial membrane potential in living cells by fluorescence microscopy J Cell Biol 1981 88 3 526 535 10.1083/jcb.88.3.526 6783667 PMC2112765

[B21] Modica-Napolitano JS Aprille JR Basis for the selective cytotoxicity of rhodamine 123 Cancer Res 1987 47 16 4361 4365 2886218

[B22] Elbaz HA Stueckle TA Tse W Rojanasakul Y Dinu CZ Digitoxin and its analogs as novel cancer therapeutics Exp Hematol Oncol 2012 1 1 4 9 10.1186/2162-3619-1-4 23210930 PMC3506989

[B23] Wang K Wei G Liu D CD19: a biomarker for B cell development, lymphoma diagnosis and therapy Exp Hematol Oncol 2012 1 1 36 42 10.1186/2162-3619-1-36 23210908 PMC3520838

[B24] Weiß L Efferth T Polo-like kinase 1 as target for cancer therapy Exp Hematol Oncol 2012 1 1 38 43 10.1186/2162-3619-1-38 23227884 PMC3533518

[B25] Banjerdpongchai R Kongtawelert P Khantamat O Srisomsap C Chokchaichamnankit D Subhasitanont P Svasti J Mitochondrial and endoplasmic reticulum stress pathways cooperate in zearalenone-induced apoptosis of human leukemic cells J Hematol Oncol 2010 3 50 65 10.1186/1756-8722-3-50 21190589 PMC3018374

[B26] Sosin AM Burger AM Siddiqi A Abrams J Mohammad RM Al-Katib AM HDM2 antagonist MI-219 (spiro-oxindole), but not Nutlin-3 (cis-imidazoline), regulates p53 through enhanced HDM2 autoubiquitination and degradation in human malignant B-cell lymphomas J Hematol Oncol 2012 5 57 74 10.1186/1756-8722-5-57 22989009 PMC3473265

[B27] Arismendi-Morillo G Electron microscopy morphology of the mitochondrial network in human cancer Int J Biochem Cell Biol 2009 41 10 2062 2068 10.1016/j.biocel.2009.02.002 19703662

[B28] Karbowski M Youle RJ Dynamics of mitochondrial morphology in healthy cells and during apoptosis Cell Death Differ 2003 10 8 870 880 10.1038/sj.cdd.4401260 12867994

[B29] Carré M André N Carles G Borghi H Brichese L Briand C Braguer D Tubulin is an inherent component of mitochondrial membranes that interacts with the voltage-dependent anion channel J Biol Chem 2002 277 37 33664 33669 10.1074/jbc.M203834200 12087096

[B30] Zamzami N Kroemer G The mitochondrion in apoptosis: how Pandora’s box opens Nat Rev Mol Cell Biol 2001 2 1 67 71 10.1038/35048073 11413468

[B31] Bommareddy A Hahm ER Xiao D Powolny AA Fisher AL Jiang Y Singh SV Atg5 regulates phenethyl isothiocyanate-induced autophagic and apoptotic cell death in human prostate cancer cells Cancer Res 2009 69 8 3704 3712 10.1158/0008-5472.CAN-08-4344 19336571 PMC2669844

[B32] Hackenbrock CR Energy-linked ultrastructural transformations in isolated liver mitochondria and mitoplasts. Preservation of configurations by freeze-cleaving compared to chemical fixation J Cell Biol 1972 53 2 450 465 10.1083/jcb.53.2.450 4554366 PMC2108731

[B33] Perkins GA Ellisman MH Mitochondrial configurations in peripheral nerve suggest differential ATP production J Struct Biol 2011 173 1 117 127 10.1016/j.jsb.2010.06.017 20600951 PMC3078762

[B34] Suhr ST Chang EA Tjong J Alcasid N Perkins GA Goissis MD Ellisman MH Perez GI Cibelli JB Mitochondrial rejuvenation after induced pluripotency PLoS One 2010 5 11 e14095 10.1371/journal.pone.0014095 21124794 PMC2991355

[B35] De Gendt K Denolet E Willems A Daniels VW Clinckemalie L Denayer S Wilkinson MF Claessens F Swinnen JV Verhoeven G Expression of tubb3, a beta-tubulin isotype, is regulated by androgens in mouse and rat sertoli cells Biol Reprod 2011 85 5 934 945 10.1095/biolreprod.110.090704 21734264 PMC4480425

[B36] Lu QY Lin XH Feng J Zhao XM Gallagher R Lee MY Chiao JW Liu DL Phenylhexyl isothiocyanate has dual function as histone deacetylase inhibitor and hypomethylating agent and can inhibit myeloma cell growth by targeting critical pathways J Hematol Oncol 2008 1 6 15 10.1186/1756-8722-1-6 18577263 PMC2438442

[B37] Cicchillitti L Penci R Di Michele M Filippetti F Rotilio D Donati MB Scambia G Ferlini C Proteomic characterization of cytoskeletal and mitochondrial class III beta-tubulin Mol Cancer Ther 2008 7 7 2070 2079 10.1158/1535-7163.MCT-07-2370 18645017

